# No late effects of growth hormone exposure on ventricular mass and function in patients with Turner's syndrome

**DOI:** 10.1186/1532-429X-15-S1-P271

**Published:** 2013-01-30

**Authors:** Rosica Panayotova, Rachael Brooks, Alexander Borg, Christopher A Miller, Matthias Schmitt

**Affiliations:** 1 University Hospital of South Manchester, Manchester, UK; 2 University of Manchester, Manchester, UK

## Background

Patients with Turner's syndrome have increased cardiovascular (CV) morbidity and mortality, potentially over and above that explained by the higher rate of both congenital heart disease and associated endocrine disorders and hypertension. GH administration to maximise adult height is a well-established treatment form but may affect cardiovascular status, ventricular mechanics and myocardial function. Furthermore, given the adverse CV effects of supra-physiological GH levels associated with acromegaly and in the field of sports doping, concern has been voiced about potential risks associated with this form of treatment.

CMR based strain derivatives offer advanced analysis of myocardial deformation and function. Such technologies are thought to increase sensitivity and may allow detection of "pre-clinical" disease not apparent by assessment of conventional parameters such as ventricular volumes and ejection fraction (EF).

## Methods

52 patients with Turner syndrome underwent a comprehensive CV CMR study. Patients with structural heart disease such as aortic coarctation, haemodynamically important valve disease, previous myocardial infarction or cardiac surgery were excluded. Of the remaining 35 adult patients 14 had a history of previous exposure to GH (GH +ve), 21 had no history of GH treatment (GH -ve).

Assessment of ventricular mass, volumes and derived EF was performed by means of signal intensity thresholding based detailed endocardial contouring using commercial software (CMRtoolsTM, Cardiovascular Imaging Solutions Ltd, UK). LV/RV longitudinal and LV circumferential strain was analysed using an endocardial feature tracking software package (2D-CPA MR, Diogenes®, TomTec Imaging Systems GmbH, Germany).

## Results

The demographic and haemodynamic data of both groups is summarised in Table 1. The mean age of GH initiation was 10 ± 4.1 years, with a mean duration of treatment of 3.7 years. Ventricular volumes and performance parameters are summarised in Table 2. Conventional resting volumetric measurements and performance indices, as well as biventricular strain measurements, were within normal limits and did not differ significantly between groups.

**Table 1:** Demographic and haemodynamic parameter data in Turner's syndrome women with a positive and negative history of growth hormone exposure. Values are displayed as means ± SD.

	GH +ve (n=14)	GH -ve (n=21)	*P* value
Age (yrs)	31.4 ± 9.8	37.4 ± 7.6	0.05

Weight (kg)	64.9 ± 16.8	69.0 ± 16.2	0.48

Height (m)	1.48 ± 0.07	1.48 ± 0.08	0.83

BMI	29.0 ± 6.5	31.5 ± 6.4	0.28

BSA (m^2^)	1.6 ± 0.2	1.6 ± 0.05	0.62

Heart Rate (bpm)	83 ± 15	84 ± 14	0.89

Blood pressure (mmHg) systolic diastolic	116 ± 20	118 ± 17	0.67
	
	73 ± 12	77 ± 12	0.31

**Table 2:** Volumetric and strain analysis data

	GH +ve (n=14)	GH -ve (n=21)	*P* value
Indexed LV EDV (ml/m^2^)	68.9 ± 9.3	63.6 ± 11.7	0.22

Indexed LV ESV (ml/m^2^)	23.1 ± 6.1	18.8 ± 6.7	0.10

LVEF (%)	67 ± 6.0	71 ± 6.8	0.16

LV mass (g/m^2^)	44 ± 7.3	42 ± 7.6	0.98

TAPSE (cm)	2.2 ± 0.5	2.1 ± 0.3	0.33

Longitudinal LV strain (%)	-17.7 ± 1.9	-18.2 ± 4.2	0.69

Longitudinal RV strain (%)	-28.7 ± 5.5	-25.3 ± 6.6	0.12

Circumferential LV strain (%)	-27.8 ± 4.5	-27.7 ± 5.5	0.96

## Conclusions

Childhood GH exposure appears to have no detectable detrimental late effects on LV/RV ventricular performance. This study therefore provides further reassurance regarding the long term safety of childhood GH administration in patients with Turner's syndrome.

## Funding

None.

